# Bilateral Emphysematous Pyelonephritis in a Retroviral Disease Patient

**DOI:** 10.7759/cureus.77358

**Published:** 2025-01-13

**Authors:** Ifiok Umana, Olutayo Osunaiye, Jila Bem, Samaila Shuaibu

**Affiliations:** 1 Urology Division, Jos University Teaching Hospital, Jos, NGA

**Keywords:** antibiotics, diabetes, emphysematous pyelonephritis, human immunodeficiency virus, renal parenchymal gas

## Abstract

Emphysematous pyelonephritis (EPN) is a rare infection characterized by the presence of gas in the renal parenchyma and surrounding tissues. This condition is frequently seen in individuals with diabetes and is rarely observed in patients with human immunodeficiency virus (HIV) infection.

A 41-year-old retroviral disease (RVD)-positive female patient on highly active antiretroviral therapy (HAART) presented with features of pyelonephritis. An abdominopelvic non-contrast CT scan revealed gas within the renal parenchyma and collecting systems of both kidneys, establishing the diagnosis of EPN. The patient was treated successfully with oral levofloxacin, which upon a repeat CT showed clearance of the gas pockets.

Emphysematous pyelonephritis is a rare but potentially life-threatening infection of the kidney, commonly occurring in diabetics and rarely found in HIV patients. In selected cases, this condition can be treated effectively on an outpatient basis with oral antibiotics alone.

## Introduction

Emphysematous pyelonephritis (EPN) is a urologic emergency characterized by an acute, necrotizing parenchymal and perirenal infection resulting from gas-forming uropathogens [1]. This condition is characterized by fever, vomiting, and flank pain, representing the classical triad [2]. Additionally, pneumaturia and hematuria may be present.

Emphysematous pyelonephritis predominantly occurs in diabetic patients, accounting for up to 95% of cases [3]. It can also be precipitated by various conditions, including urinary tract obstruction from ureteric stones and papillary necrosis, as well as malignancies, drug overuse, neurogenic bladder, anatomic deformities of the urinary system, etc. Emphysematous pyelonephritis is life-threatening, with a reported mortality rate approaching 18.8% [1] and 43% [4] in some series.

The pathological classification of EPN includes four classes, Class 1 to 4, and treatment approaches are stratified accordingly.

There are limited instances where bilateral EPN has been successfully managed solely with medical management.

In this report, we detailed the case of a patient who is positive for retroviral disease (RVD) on highly active antiretroviral therapy (HAART) who presented with bilateral EPN and was successfully treated with antibiotics alone.

## Case presentation

A 41-year-old female patient with a diagnosis of RVD who has been on HAART for seven years presented to the surgical outpatient clinic complaining of a four-year history of bilateral flank pain, described as dull, aching, and non-radiating. The patient also reported a history of pneumaturia, hematuria, fever, and vomiting. There was no history of diarrhea, fecaluria, or features suggestive of inflammatory bowel disease. No history of necroturia, passage of stones in the urine, weight loss, or features suggestive of lower urinary tract symptoms (LUTS). However, she was not a known diabetic or alcoholic and had no relevant past medical history.

Upon examination, vital signs at presentation were normal, and a general physical examination showed a healthy-looking young woman; the head and neck, chest, abdomen, and limb findings were unremarkable.

**Figure 1:**
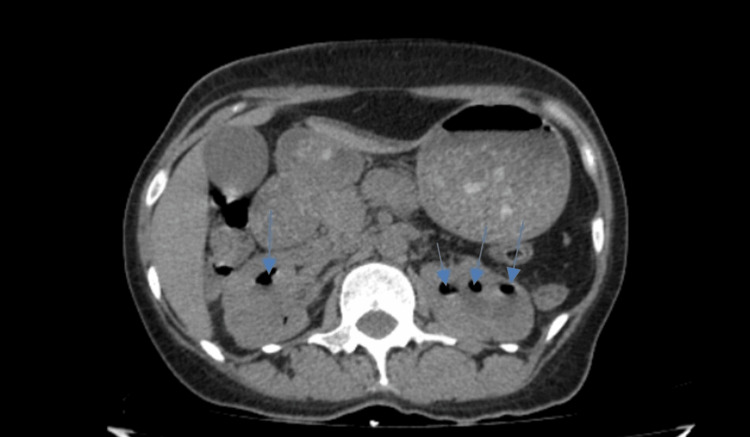
The CT scan shows bilateral emphysematous pyelonephritis before antibiotic treatment

Laboratory test results indicated two to three red blood cells (RBC) per high-power field (hpf) on urinalysis; urine microscopy, culture, and sensitivity tests yielded a growth of *Klebsiella *spp. sensitive to levofloxacin. The biochemical profile showed elevated levels of urea and creatinine. Hematological parameters were within normal limits, and the other relevant investigations, including fasting blood glucose (FBG) and cluster of differentiation 4 (CD4) count, are shown in Table 1. The patient was referred for a non-contrast abdominopelvic CT scan, which demonstrated multiple air collections within the parenchyma and collecting systems of both kidneys, along with a hyperdense filling defect in the gallbladder, with no features suggestive of renal calculi (Figure 1).

**Table 1:** The patient's laboratory values FBG: fasting blood glucose; CD4: cluster of differentiation 4

	Before treatment (µmol/l)	After treatment (µmol/l)	Reference range (µmol/l)
Urea	7.4	7.4	2.5 – 8.0
Creatinine	204	250	72 – 126
FBG	6.4		3.3 – 5.8 mmol/l
CD4 count	652		500 – 1500 cells/µl

A diagnosis of bilateral EPN (Class 4) was made based on the clinical features and imaging findings. She was counseled on the diagnosis and modality of treatment. The patient was commenced on oral levofloxacin with continued observation.

**Figure 2:**
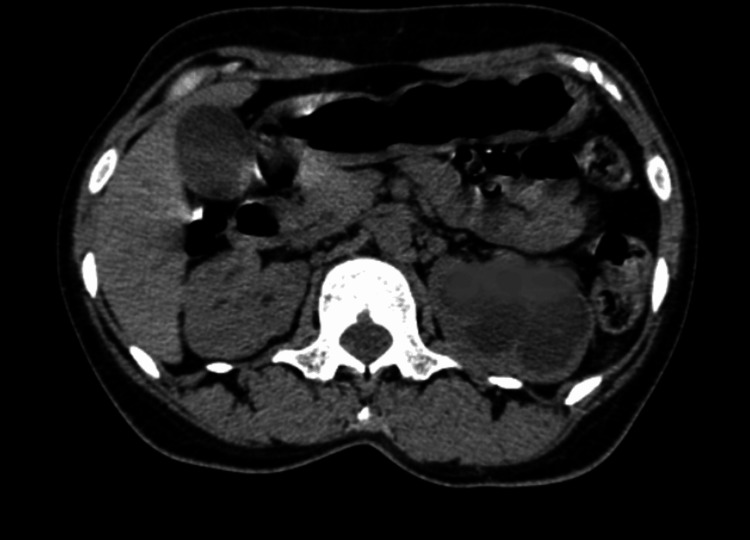
The CT scan shows complete resolution of right renal emphysematous pyelonephritis after oral antibiotics

At follow-up, symptoms significantly improved; however, the renal function showed no improvement. Following antibiotic therapy with oral levofloxacin, a repeat CT showed complete resolution of the EPN (Figure 2). 

The patient is being followed up at the outpatient clinic and reports no abnormal symptoms.

## Discussion

Emphysematous pyelonephritis is an acute necrotizing infection of the renal parenchyma and its surrounding areas, characterized by the presence of gas in the renal parenchyma, collecting system, or perinephric tissue [5]. It was first described by Kelly and MacCallum [6] in 1898, although Schultz and Klorfein [7] first used the term "emphysematous pyelonephritis" in 1962.

This condition is predominantly seen in diabetic patients, accounting for up to 95% of cases, with a 25%-40% risk of developing EPN secondary to urinary tract obstruction [3]. Other associated risk factors include immunosuppression, alcoholism, drug abuse, malignancies, neurogenic bladder, and anatomical abnormalities of the urinary system. Our index case was retroviral disease positive.

The incidence of EPN in HIV-infected patients is considered rare, with few documented cases [8-10].

Reports indicate a demographic preponderance of the disease among females (41:7 [5], 3:1 [11,12], 43:3 [13]), while children do not exhibit the disease. Emphysematous pyelonephritis is more likely to affect the left kidney than the right (60% vs. 35%), with bilateral involvement in 5% of cases [11].

Emphysematous pyelonephritis represents a life-threatening condition, with mortality rates ranging from 18.8% [1] to 43% [4], and potentially as high as 90% [14]. Patients typically present with the classical triad of fever, vomiting, and flank pain [4]. Other symptoms may include pneumaturia, hematuria, and pyuria. Our patient presented with recurrent bilateral flank pain, hematuria, pneumaturia, low-grade fever, and vomiting.

The pathogenesis of EPN is attributed to the high glucose concentration serving as a culture medium for uropathogens; however, in non-diabetic patients, urinary albumin and tissue protein have been said to be the source of fermentation [15]. Other mechanisms proposed include impaired vascular supply, impaired immune system, and ureteral obstruction [11,16]. It is still unknown why some people develop emphysematous pyelonephritis, while others simply develop a conventional urinary tract infection.

*Escherichia coli* is the most common causative organism in about 70% of cases [1], followed by *Klebsiella*, *Proteus*, and *Pseudomonas *species. Coagulase-negative *Staphylococcus* and Group D *Streptococcus *have also been implicated. In our patient’s case, urine *Klebsiella *spp. was cultured from the urine.

Establishing the diagnosis of EPN requires the use of imaging modalities, with CT being the preferred diagnostic modality. Other investigations of importance include urine MCS and renal function tests.

Over the years, pathological classifications based on imaging modalities have evolved [5,17,18]. 

Michaelis et al. [17] in 1984 were the first to classify EPN based on the findings of plain abdominal films of the kidney, ureter, and bladder (KUB) and intravenous pyelogram (IVP).

Wan et al. [18] in 1996 classified EPN based on CT findings into Types I and II, while in 2000, Huang and Tseng [5] published a different classification system, which was also based on CT findings but had more details than the previous one (Table 2).

**Table 2:** Huang and Tseng's classification of emphysematous pyelonephritis (EPN) Adapted from [5].

Class	Description
Class 1 (mild)	Gas within the renal collecting system only
Class 2 (mild)	Gas in the renal parenchyma
Class 3A (severe)	Perinephric gas or abscess formation
Class 3B (severe)	Perirenal gas or abscess formation
Class 4 (severe)	Bilateral EPN or solitary kidney with EPN

Prognostic factors adversely affecting outcomes include diabetes mellitus, thrombocytopenia, elevated serum creatinine, altered sensorium, and shock [5]. Notably, our patient did not manifest any of these poor prognostic factors.

**Figure 3:**
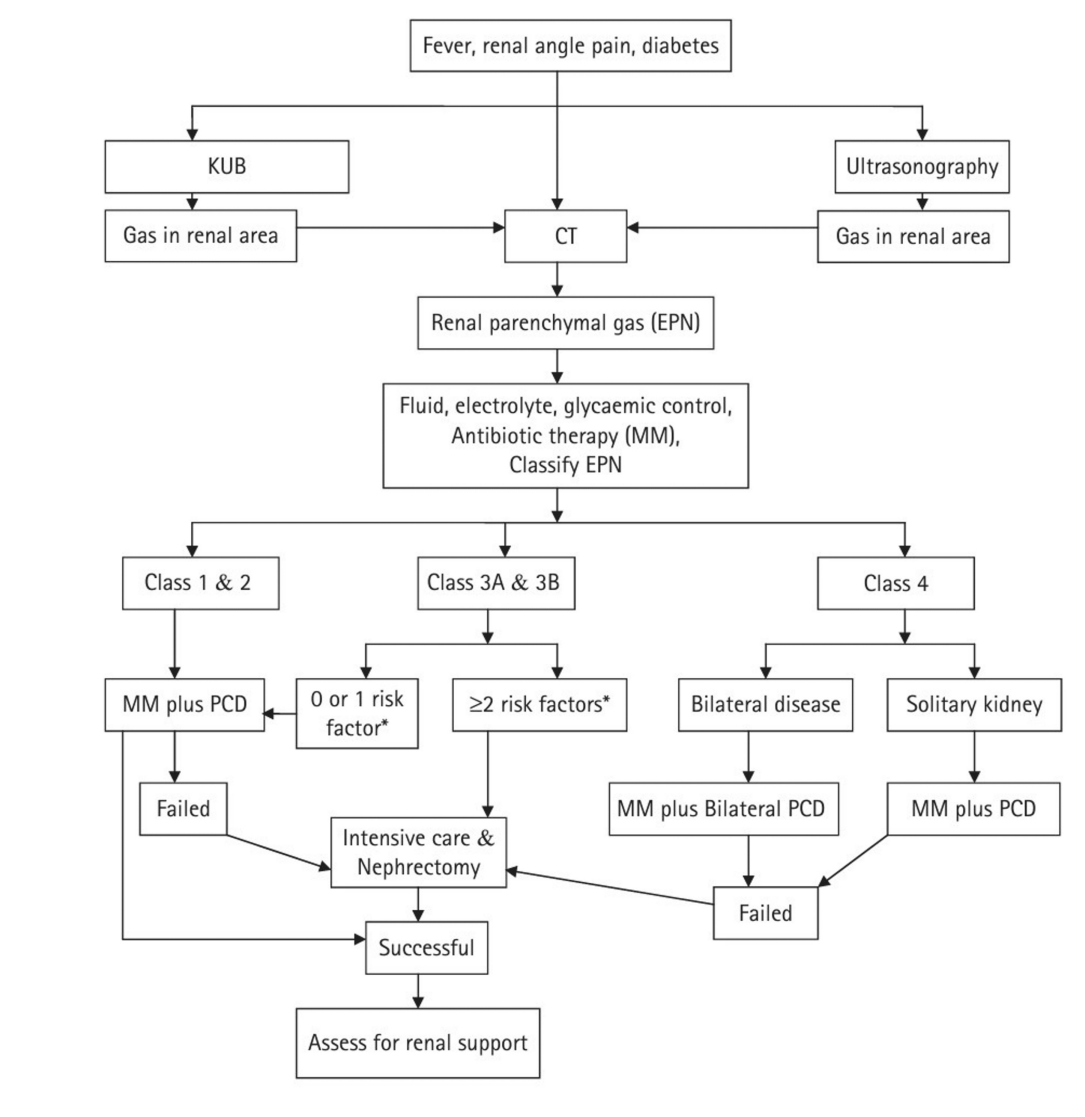
Management algorithm for EPN based on clinico-radiologic classification by Huang and Tseng *Risk factors: diabetes, thrombocytopenia, acute renal failure, altered level of consciousness, shock. EPN: emphysematous pyelonephritis; KUB: kidney, ureter, and bladder; MM: medical management; PCD: percutaneous drainage Source: [3]; reproduced with permission from the authors.

The treatment options are based on the pathological classification of the disease and the presence of any poor prognostic factors (Figure 3).

The gold standard for managing emphysematous pyelonephritis includes either medical management (MM) with antibiotics or percutaneous drainage (PCD) with MM with or without nephrectomy [19].

Numerous series have documented positive outcomes with conservative management utilizing antibiotics alone in patients with bilateral EPN [20]. 

## Conclusions

Emphysematous pyelonephritis is a rare urinary tract infection characterized by gas in the renal parenchyma and surrounding tissues. It is rarely seen among HIV patients. It is associated with significant mortality, especially in patients with poor prognostic factors. Abdominal CT is the imaging modality of choice since early identification can be decisive in disease prognostication. Treatment options are determined based on the class of the disease.

In our patient, who was RVD-positive on HAART with Class 4 EPN, the conservative option was deemed suitable due to her overall clinical status and prognostic factors. This case report illustrates that conservative management can be a viable and acceptable alternative to surgical intervention, even in instances of bilateral EPN.
